# Novel *CHP1* mutation in autosomal-recessive cerebellar ataxia: autopsy features of two siblings

**DOI:** 10.1186/s40478-020-01008-2

**Published:** 2020-08-12

**Authors:** Rie Saito, Norikazu Hara, Mari Tada, Yoshiaki Honma, Akinori Miyashita, Osamu Onodera, Takeshi Ikeuchi, Akiyoshi Kakita

**Affiliations:** 1 Department of Pathology, Brain Research Institute, Niigata University, 1-757 Asahimachi, Chuo-ku, Niigata, 951-8585 Japan; 2 Department of Molecular Genetics, Brain Research Institute, Niigata University, 1-757 Asahimachi, Chuo-ku, Niigata, 951-8585 Japan; 3 Department of Neurology, Sado General Hospital, 161 Chigusa, Sado, Niigata, 952-1209 Japan; 4 Department of Neurology, Brain Research Institute, Niigata University, 1-757 Asahimachi, Chuo-ku, Niigata, 951-8585 Japan

**Keywords:** CHP1, NHE1, Autosomal recessive cerebellar ataxia, Middle-aged onset, Neuropathology

(To the editor)

Recent genetic studies have led to the discovery of many novel causative genes of autosomal recessive cerebellar ataxias (ARCAs), which represent an extensive group of clinically and genetically heterogeneous neurodegenerative disorders that are manifested mostly in children [1]. For example, biallelic mutation in *Calcineurin homologous protein-1* (*CHP1*) has been identified recently in two siblings of a consanguineous family showing cerebellar atrophy, spastic ataxia, motor neuropathy, intellectual disability, and slow ocular saccades (ARCA-*CHP1*) [2]. CHP1 serves as an essential cofactor of NHE1 (Na^+^ / H^+^ exchanger 1) which is a major regulator of intracellular ions and pH homeostasis. In vitro and in vivo studies of ARCA-*CHP1* have shown that alteration of CHP1 and NHE1 expression could affect crucially ARCA pathomechanisms [2, 3]. However, the neuropathologic features and alteration of CHP1 and NHE1 expression remain unknown in patients with ARCA-*CHP1*. Here we investigated the clinicopathologic and biochemical features of two autopsied siblings with ARCA harboring a novel homozygous missense mutation in *CHP1* identified through whole-exome sequencing (WES).

Two siblings (patients 1 and 2) developed cerebellar ataxic gait and speech at the ages of 30 and 56 years, respectively, followed by cognitive decline, pyramidal signs, loss of deep tendon reflexes and hearing loss. In patients 1 and 2, brain CT images revealed diffuse cerebellar atrophy (Supplementary Fig. 1). Their clinical features were summarized in Table 1, and described in detail in Additional file 1.

**Table 1:** Clinical features of patients with ARCA-*CHP1*

	Present report		Mendoza-Ferreira, et al. [2]	
	Patient 1	Patient 2	Reported siblings	
**Consanguinity**	+		+	
**Age at onset (age/sex)**	30 y/M	56 y/F	12 mon/F	5 y/M
**Disease duration (y)**	36	20	> 25	> 15
**Initial symptom**	Gait instability	Gait instability	Gait instability	Frequent falls
**Cerebellar signs**				
Gait instability	+	+	+	+
Dysarthria	+	+	–	n.a.
Ocular dysmetria	+	+	+	+
**Pyramidal signs**				
Muscle weakness	–	–	+	+
Deep tendon reflexes	Hypo-loss	Hypo-loss	Hyper	Hyper
Babinski signs	+	+	+	+
**Neuropathy**				
Loss of superficial sense	–	–	–	n.a.
Loss of vibration sense	+	–	–	+
**Others**				
Mild intellectual disability	–	+	+	+
Cognitive decline	+	+	n.a.	n.a.
Hearing loss	+	+	–	n.a.
Hypogonadism	–	n.a.	+	n.a.
**Neuroimaging**				
Cerebellar atrophy (vermis/hemisphere)	+/+	+/+	−/−	+/−

(+) and (−), presence and absence, respectively; n.a., not available

The histopathologic features of the nervous system in patients 1 and 2 were quite similar, being characterized by marked degeneration of the cerebellum and dorsal column pathway. These changes were more severe in the patient with younger disease onset. Atrophy of the cerebellar hemispheres was more severe than that of the vermis (Fig. 1a). Microscopically, severe loss of Purkinje cells with Bergman gliosis, being more prominent in the cerebellar hemisphere than in the vermis, was evident (Fig. 1b and c). Immunoreactivity of calbindin-D28k in the remaining Purkinje cells was decreased (Fig. 1d and Fig. 2j), whereas that of parvalbumin in stellate cells and basket cell was relatively preserved (Fig. 1e). In the dentate nucleus, although the neurons were shrunken, neuronal loss was not obvious (Fig. 1f). Regarding the cerebellar afferent system, degeneration of the inferior olivary nuclei and pontine nucleus was unremarkable. No neuronal loss or focal gliosis was observed in the other regions of the brainstem and cerebrum, except for moderate neuronal loss (Fig. 1g) and gliosis (Fig. 1h) in layers II and III of the frontal cortex. There were no expanded polyglutamine-positive or ubiquitinated inclusions in the affected areas. The brains showed no pathological features suggestive complications arising from Alzheimer’s disease or Parkinson’s disease. The spinal cord and dorsal roots were atrophic (Fig. 1i). The gracile fasciculus showed loss of myelinated fibers extending from the cervical to the lumbar level (Fig. 1j), and the dorsal root ganglia showed severe loss of ganglion cells (Fig. 1k). Severe loss of myelinated fibers in the sural nerve was also evident (Fig. 1l).

**Fig. 1:**
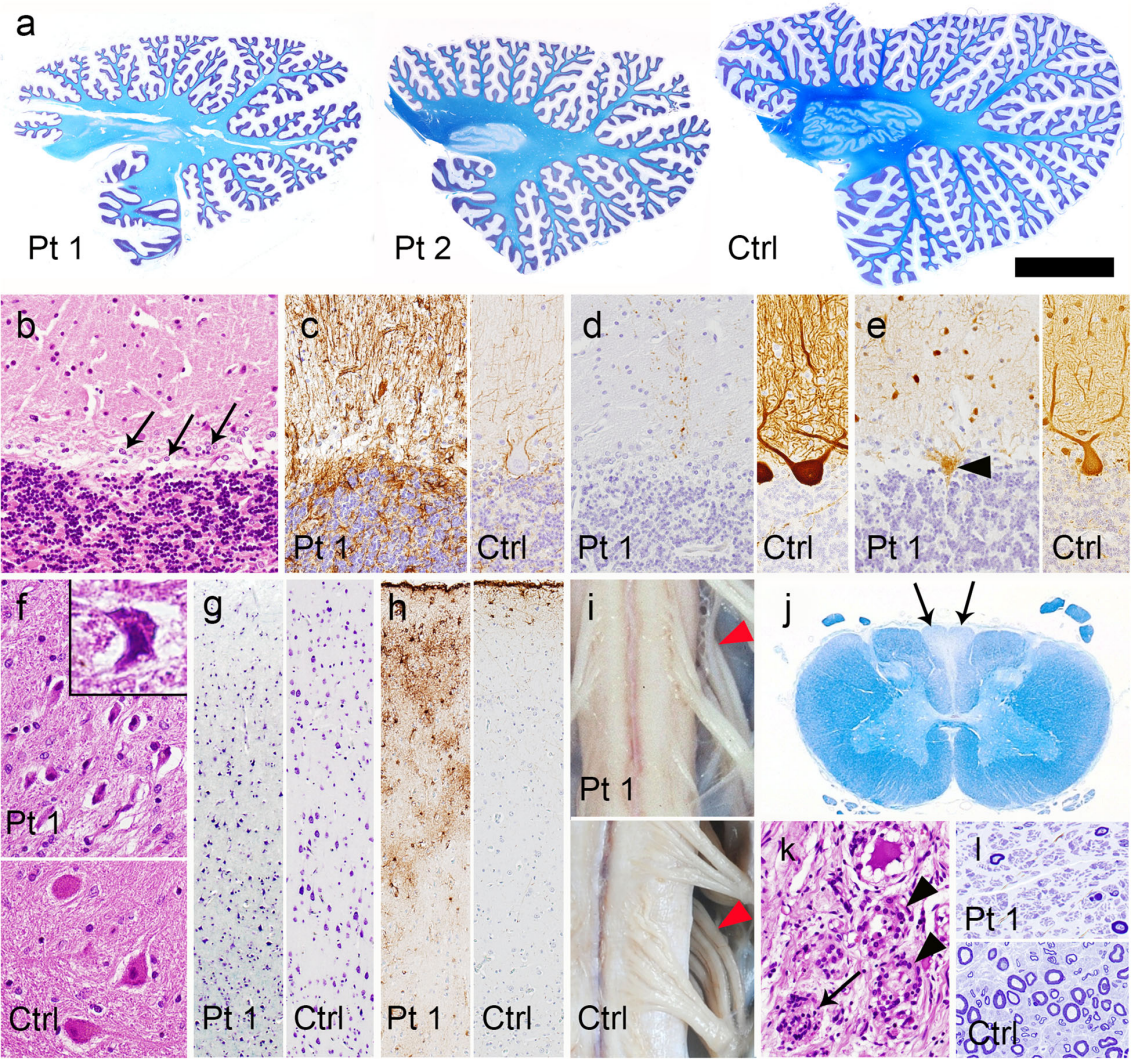
Neuropathologic findings. **a** Sagittal sections of the cerebellum showing diffuse atrophy of the folia and thinning of the dentate nucleus. The superior cerebellar peduncles are spared. Klüver-Barrera staining. **b** Loss of Purkinje cells and Bergmann gliosis (*arrows*) in the hemisphere. HE staining. **c** Bergmann gliosis is more evident by GFAP immunohistochemistry (IHC). **d** Decreased immunoreactivity of calbindin-D28k in the cerebellar cortex. The cell body and dendrites of a Purkinje cell are strongly stained in the control brain. Calbindin-D28k-IHC. **e** Retained parvalbumin immunoreactivity in the remaining basket and stellate cells in the cerebellar molecular layer, and an empty basket (*arrowhead*). The cell body and neurites of these interneurons in the cerebellar molecular layer, and those of a Purkinje cell are also stained in the control brain. Parvalbumin-IHC. **f** Although neurons in the dentate nucleus are shrunken (inset), their number is preserved. HE staining. **g** Moderate loss and shrinkage of neurons observed using Klüver-Barrera staining, and (**h**) gliosis detected by GFAP-IHC in the frontal cortex. **i** Atrophy of the cervical cord and posterior roots (*arrowheads*). **j** Atrophy and myelin pallor of the gracile fasciculus (*arrows*). Klüver-Barrera staining. **k** Loss of ganglion cells with a Nageotte nodule (*arrow*) and macrophage infiltration into the spaces where ganglion cells have been lost (*arrowheads*) in the dorsal root ganglion of the lumbar spinal cord. HE staining. (**l**) Severe loss of myelinated fibers in the sural nerve. Toluidine blue stain. Patient 1. Ctrl, control; Pt, patient. Bars = 1 cm in **a**; 8 mm in **i**; 300 μm in **g**, **h**, **j**; 100 μm in **b-f**, **k**; 50 μm in **l**.

**Fig. 2:**
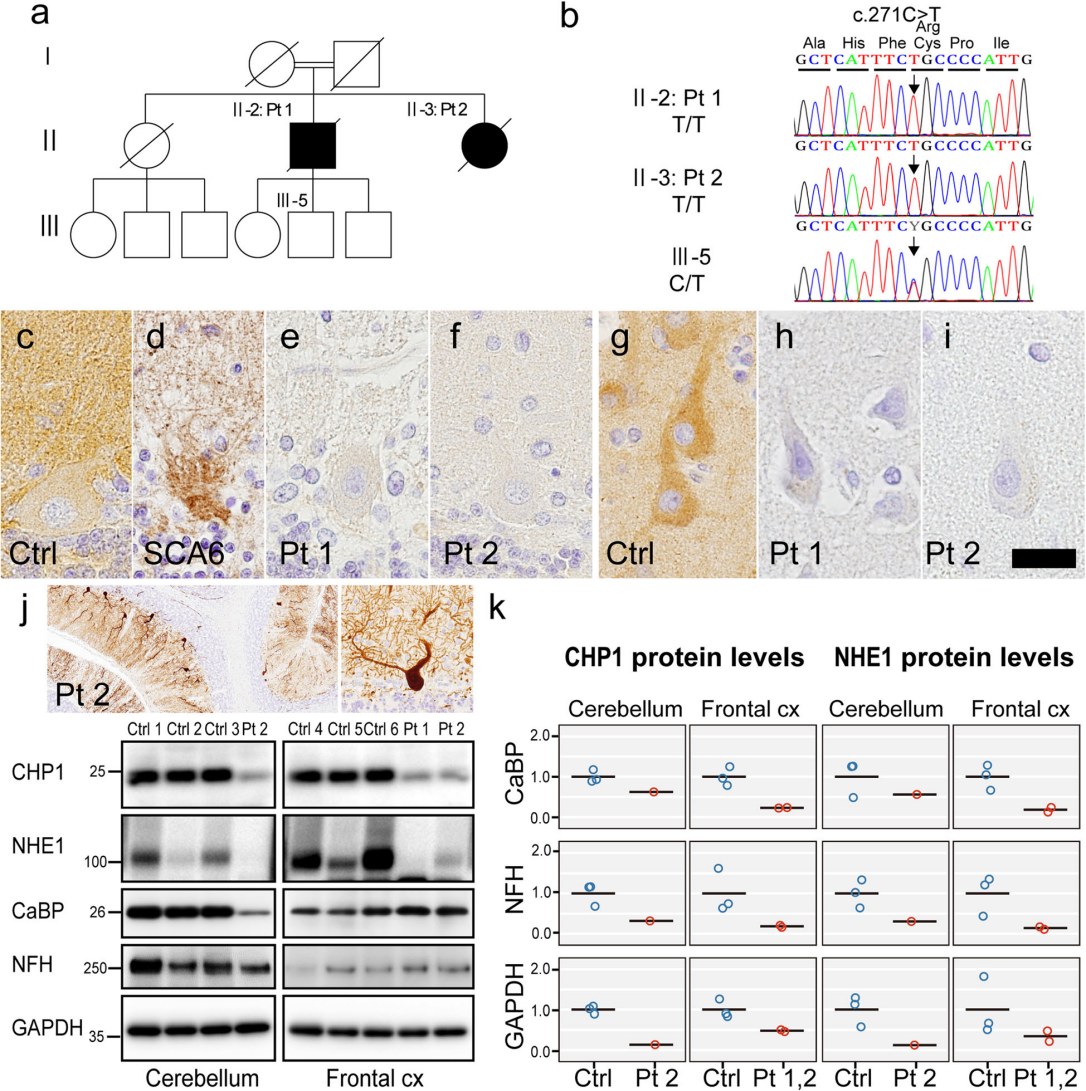
Expression of CHP1 and NHE1 in autopsied tissue. **a** Pedigree of the family. **b** Electrophoretograms showing sequencing of the c.271C > T *CHP1* mutation. Patients 1 and 2 harbor a homozygous mutation and III-5 harbors a heterozygous mutation. **c-i** CHP1 immunohistochemistry. Images of the Purkinje cell layer (**c-f**) and frontal cortex (**g-i**). Positive reactivity is evident in the membrane and cytoplasm of the Purkinje cell and neuropil in the control (**c**), and empty basket in the disease control of spinocerebellar ataxia type 6 (**d**), but absent in the patients 1 and 2 (**e, f**). CHP1 immunoreactivity in the neuronal cytoplasm and neuropil is evident in the control (**g**), but absent in the patients 1 and 2 (**h, i**). **j** Moderate loss of calbindin-D28k (CaBP)-immunoreactive Purkinje cells and their dendrites in the cerebellum of patient 2 (*upper left panel*). Magnified image of a Purkinje cell with retained expression of CaBP (*upper right panel*). Western blotting of autopsied brain samples taken from the cerebellum and frontal cortex using antibodies for CHP1 and NHE1, and also CaBP, neurofilament-H (NFH) and GAPDH as loading controls (*lower panel*). Note the moderate reduction of CaBP expression in the cerebellum of patient 2, being consistent with the moderate loss of Purkinje cells observed with CaBP-IHC. **k** Relative levels of expression of CHP1 and NHE1 proteins. The levels were determined by normalization against CaBP, NFH and GAPDH. The bar shows the mean value under each condition. The protein levels of both CHP1 and NHE1 are reduced in the patients relative to the controls. Ctrl, control; SCA6, a woman aged 76 years with a disease duration of 19 years; Pt, patient; CaBP, calbindin-D28k; NFH, neurofilament-H. Bars = 350 μm in **j** (***left panel***)**;** 80 μm in **j** (***right panel***); 30 μm in **g-i**; 20 μm in **c-f**

WES analysis uncovered 6672 variants that segregated in an autosomal-recessive manner, in which seven candidates were prioritized (Table 2 and Additional file 1). Among them, we focused on a homozygous missense variant, p.Arg91Cys (c.271C > T), in *CHP1*. The variant has not been found in publicly available databases and exhibited the highest CADD score [4] – 32.0 – of all the candidates, indicating a highly predictively-damaging effect, and considered “likely pathogenic” based on the ACMG guidelines [5]. The variant was confirmed by Sanger sequencing (Fig. 2a and b). CHP1 immunoreactivity was detected in the membrane and cytoplasm of neurons and the neuropil in controls, but was completely lost in the cerebellar and cerebral cortex of the patients (Fig. 2c-i). Indeed, immunoblot analysis revealed that expression of CHP1 protein in the patients was reduced in the cerebellum by 80% and in the frontal cortex by 60% relative to the controls. Moreover, we confirmed a severe reduction of NHE1 protein in those tissues (Fig. 2j and k). The levels of CHP1 and NHE1 expression remained consistently decreased when normalized against those of calbindin-D28k or neurofilament. These results indicated that the reduction of CHP1 and NHE1 was not due to loss of Purkinje cells or other neurons. Details of methods are in Additional file 1.

**Table 2:** Profiles of candidate variants segregating in the studied patients

Chr	Start	End	dbSNP	Ref	Alt	Gene	Transcript	Exon	Codon change	Amino acid change	Impact	Max_aaf_all (%)	ToMMoaaf (%)	CADD	Cbll
15	41,555,002	41,555,003	–	C	T	*CHP1*	NM_007236.4	4/7	c.271C > T	p.R91C	missense	–	–	32.0	30.08
6	31,935,498	31,935,499	rs765832592	C	T	*SKIV2L*	NM_006929.4	22/28	c.2591C > T	p.S864L	missense	0.059	0.050	17.6	44.13
6	32,148,942	32,148,943	–	C	T	*AGER*	NM_001206929.1.3	11/11	c.1240G > A	p.E414K	missense	–	0.010	12.1	26.89
15	34,048,529	34,048,530	rs766762265	A	G	*RYR3*	NM_001036.4	59/104	c.8539A > G	p.T2847A	missense	0.307	0.170	9.7	4.68
6	27,368,455	27,368,456	rs749223275	T	G	*ZNF391*	NM_001076781.2	3/3	c.307 T > G	p.F103V	missense	0.058	0.040	8.7	4.63
15	43,816,412	43,816,413	rs201851388	C	A	*MAP1A*	NM_002373.5	4/6	c.2742C > A	p.D914E	missense	0.255	0.270	8.0	136.43
6	28,253,358	28,253,359	rs760010326	A	C	*PGBD1*	NM_001184743.1	3/7	c.428A > C	p.H143P	missense	0.058	0.040	5.1	17.49

Seven segregated candidate variants causing protein alteration and showing a low allele frequency of < 1% and gene expression > 1 median transcripts per kilobase million (TPM) in the cerebellum. Variants are ordered according to the CADD score. None of the other six candidates except for *CHP1* mutation demonstrated a causal link to ARCAs. Therefore, we considered that the novel homozygous missense variant *CHP1* p.Arg91Cys had been responsible for the ataxic phenotype of the two present cases. Variants were annotated using *snpEff* 4.3i and *gemini* 0.19.1. The genomic positions of the variants are based on hg19, and *Start* and *End* represent the 0-based and 1-based genomic position, respectively. *Impact* shows the biological consequence of the most severely affected transcript. *Max_aaf_all* shows the maximum alternate allele frequency in each population from the 1000 Genomes Project, Exome Sequencing Project, or Exome Aggregation Consortium. *ToMMo aaf* shows the alternate allele frequency in 3554 Japanese whole genomes obtained from Tohoku University Tohoku Medical Megabank Organization. *CADD* shows the PHRED-like scaled scores of predictive deleteriousness, and typically scores of 10 or higher indicate possibly pathogenic variants. The column labeled *Cbll* represents the amount of gene expression in the cerebellum based on GTEx Portal V8. The values show the median TPM

Assuming a compound heterozygous model, we found four candidate variants located in two genes, *ZNF440* and *ZNF804A* (Supplementary Table 2). However, both variants in *ZNF440* appeared benign, because their CADD scores were lower than 10. For *ZNF804A*, we failed to find any study that had associated it with cerebellar ataxia. Accordingly, the homozygous variant in *CHP1* was considered most likely to be linked with the present phenotype based on the genetic and pathological findings.

Comparing the present patients with the reported two siblings harboring a homozygous p.Lys19del *CHP1* mutation [2], despite sharing other clinical manifestations, the age at onset differed considerably between the two families and the most significant clinical feature in the present patients was onset of ataxia in middle age and cognitive decline, in contrast to the infantile-onset ataxia and intellectual disability in the reported patients (Table 1). Such differences in the clinical features might have been a consequence of the different pathogenic variants. Indeed, in vitro experiments have revealed that the pathogenic variant p.Lys19del led to almost complete loss of the CHP1 protein [2], whereas immunoblotting in our patients demonstrated incomplete reduction of CHP1 protein in the brain tissue harboring the p.Arg91Cys *CHP1* mutation. This remaining protein expression could have resulted in the milder phenotype.

Based on our observations, the CHP1 insufficiency was presumed to have been linked to neuronal loss in the cerebellar and frontal cortex, which would have been associated with cerebellar ataxia and cognitive decline, respectively. Indeed, Chp1 deficiency in zebrafish causes cerebellar hypoplasia, movement disorder and motor axon abnormalities, which can be ameliorated by co-injection with wild-type human *CHP1* mRNA [2]. Furthermore, we demonstrated a definite reduction in the levels of CHP1 and NHE1 expression in the affected brain tissue, resembling the findings in mice with a homozygous point mutation of *chp1* [3]. Since neither the p.Arg91Cys variant nor the p.Lys19del found in the reported siblings is located in EF-hand motifs that preferentially bind to Ca^2+^ [6], a direct role of these variants in the calcium-dependent interaction between CHP1 and NHE1 would appear to be unlikely. However, the CHP1 p.Lys19del variant failed to form functional protein complexes and showed a tendency for aggregation, resulting in decreased levels of soluble CHP1 and membrane expression of NHE1 in cultured cells [2]. It can be speculated that similar mechanisms might have been operating in the present siblings.

In conclusion, our findings suggest that CHP1 insufficiency resulting from p.Arg91Cys mutation in the affected tissue might have caused loss of neurons in the cerebellum and frontal cortex mediated by the reduction of NHE1 expression. Further studies are needed to clarify the significance of *CHP1* p.Arg91Cys mutation in the context of CHP1-related neurodegeneration. Our findings broaden the clinicopathologic and pathophysiologic heterogeneity of ARCA. When encounting patients with middle-aged-onset ARCA accompanied by cognitive decline, ARCA-*CHP1* should be considered.

## Supplementary information

**Additional file 1.**

## Data Availability

The datasets used and analysed during the current study available from the corresponding author on reasonable request.

## Abbreviations

ARCA: Autosomal recessive cerebellar ataxia
*CHP1*: *Calcineurin homologous protein-1* gene
NHE1: Na^+^ / H^+^ exchanger 1; WES: whole-exome sequencing

## References

[1] Anheim M, Tranchant C, Koenig M (2012). The autosomal recessive cerebellar ataxias. N Engl J Med.

[2] Mendoza-Ferreira N, Coutelier M, Janzen E, Hosseinibarkooie S, Löhr H, Schneider S (2018). Biallelic CHP1 mutation causes human autosomal recessive ataxia by impairing NHE1 function. Neurol Genet.

[3] Liu Y, Zaun HC, Orlowski J, Ackerman SL (2013). CHP1-mediated NHE1 biosynthetic maturation is required for Purkinje cell axon homeostasis. J Neurosci.

[4] Kircher M, Witten DM, Jain P, O'Roak BJ, Cooper GM, Shendure J (2014). A general framework for estimating the relative pathogenicity of human genetic variants. Nat Genet.

[5] Richards S, Aziz N, Bale S, Bick D, Das S, Gastier-Foster J (2015). Standards and guidelines for the interpretation of sequence variants: a joint consensus recommendation of the American College of Medical Genetics and Genomics and the Association for Molecular Pathology. Genet Med.

[6] Pang T, Hisamitsu T, Mori H, Shigekawa M, Wakabayashi S (2004). Role of calcineurin B homologous protein in pH regulation by the Na+/H+ exchanger 1: tightly bound Ca2+ ions as important structural elements. Biochemistry.

