# Supplementary material for: Novel CHP1 mutation in autosomal-recessive cerebellar ataxia: autopsy features of two siblings
Source: Acta Neuropathol Commun. 2020 Aug 12;8:134. doi: 10.1186/s40478-020-01008-2 (PMC7425070; doi:10.1186/s40478-020-01008-2)
Supplement: Supplementary file 1 — Additional file 1. [file 40478_2020_1008_MOESM1_ESM.pdf]

Additional file 1

Novel *CHP1* mutation in autosomal-recessive cerebellar ataxia: autopsy features of two siblings

Rie Saito[1], Norikazu Hara[2], Mari Tada[1], Yoshiaki Honma[3], Akinori Miyashita[2], Osamu Onodera[4], Takeshi Ikeuchi[2], Akiyoshi Kakita[1]

Departments of [1]Pathology, [2]Molecular Genetics, and [4]Neurology, Brain Research Institute, Niigata University, 1 Asahimachi, Chuo-ku, Niigata 951-8585, Japan.
[3]Department of Neurology, Sado General Hospital, 161 Chigusa, Sado, Niigata 952-1209, Japan.

Correspondence to: Rie Saito, MD, PhD, or Mari Tada, MD, PhD
Department of Pathology, Brain Research Institute, Niigata University, 1-757 Asahimachi, Chuo-ku, Niigata 951-8585, Japan.
E-mail: riesaito@bri.niigata-u.ac.jp; tadamari@bri.niigata-u.ac.jp

Supplementary methods
Case presentation
Supplementary references
Supplementary figure 1: Brain CT images of patient 2
Supplementary table 1: Primary antibodies
Supplementary table 2: Profiles of compound heterozygous candidate variants segregating in the studied patients

Supplementary methods

Histopathologic analysis

The brains and spinal cords were fixed with 20% buffered formalin, and multiple tissue blocks were embedded in paraffin. Histological examination was performed on 4-μm-thick sections using hematoxylin and eosin and Klüver-Barrera staining. In addition, selected sections were immunostained with antibodies against calbindin D-28k, parvalbumin, CHP1, polyglutamine and p62 (**Supplementary table 1**).

To test the specificity of the anti-CHP1 antibody, we performed immunohistochemistry on paraffin-embedded tissue sections of the cerebellum and frontal lobe of 3 individuals without any neurological disorders (2 men aged 58 and 76 years, and a woman aged 64 years) as normal controls, and other patients with other forms of ausotomal recessive cerebellar ataxia (a man aged 72 years and a woman aged 74 years), spinocerebellar ataxia (SCA) type 3 (a man aged 71 years), SCA type 6 (a woman aged 76 years), multiple system atrophy (a man aged 68 years), paraneoplasic syndrome (a woman aged 81 years) and Altzheimer's disease (a woman aged 78 years) as disease contorls.

Antibodies against amyloid β 11-28, phosphorylated tau and phosphorylated α-synuclein were used to assess senile pathologic changes based on "ABC" score [4] and the fourth consensus report of the DLB consortium [4]. Bound antibodies were visualized by the peroxidase-polymer-based method using a Histofine Simple Stain MAX-PO kit (Nichirei, Tokyo, Japan) with diaminobenzidine as the chromogen. Immunostained sections were counterstained with hematoxylin.

To assess the sural nerve of patient 1, tissue was fixed in 2.5% glutaraldehyde in 0.125 M cacodylate buffer and embedded in epoxy resin. Semithin sections were then prepared and stained with toluidine blue.

Genetic analysis

Genomic DNA was extracted from the fresh-frozen frontal cortex (patient 1), liver (patient 2), and peripheral lymphocytes (son of patient 1). The extracted genomic DNA was used to prepare exome libraries using either a SureSelect Human All Exon V5 kit (Agilent Technologies, Santa Clara, CA, USA) for the two patients or a SureSelect Human All Exon V6 kit (Agilent Technologies) for the healthy child. The exome libraries were sequenced on Illumina HiSeq sequencers, and each library yielded more than 55,000,000 100-bp paired-end reads, allowing at least 55% mean coverage in the targeted regions.The sequenced reads were mapped to the human reference genome hg19 using *BWA-MEM* 0.7.15-r1140 [2] with default settings. The subsequent analyses—read processing, variant calling, and variant filtration—were conducted according to GATK 3.7 Best Practice recommendations [8], followed by variant annotation using *snpEff* 4.3i [1]. The resulting variant call sets were stored in an SQLite database using *gemini* 0.19.1 [6].

To identify pathogenic variants showing an autosomal recessive inheritance pattern, we

selected variants where the two affected siblings had a homozygous genotype and the unaffected child had a heterozygous genotype on the basis of the consanguinity in this pedigree. Furthermore, assuming a compound heterozygous recessive inheritance, we selected variants where the two affected siblings had a heterozygous genotype and then chose genes with two or more such variants. If the unaffected child also had two or more variants in a given gene, we excluded them. We further narrowed down candidate variants by filtering out those lacking protein alteration and showing an allele frequency of > 1% in the following publicly available databases: 1000 Genomes Project, Exome Sequencing Project, Exome Aggregation Consortium, and Tohoku University Tohoku Medical Megabank Organization. Moreover, considering that the clinicopathologic features of the present siblings were those of spinocerebellar ataxia, we excluded variants located in genes whose expression levels were quite low in the cerebellum based on GTEx Portal V8. We considered genes with greater than 1 median TPM (transcripts per kilobase million) to be "expressed" in the cerebellum. Finally, we interpreted the pathogenicity of a candidate variant based on the ACMG guidelines [7].

A candidate variant located in the *CHP1* gene was confirmed by Sanger sequencing using the following primer pair: 5'-TGAGTCCCAAAGTACCATGC-3' (forward) and 5'-CCAGTATGCTCTTGTAGATGCTTG-3' (reverse).

Western blotting

Proteins were extracted from fresh-frozen samples of the cerebellum and frontal cortex. As controls, individuals without any neurological disorders were used (cerebellum: a male aged 51 years and two females aged 60 and 68 years, frontal cortex: a male aged 79 years and two females aged 80 and 82 years). Approximately 300 mg of each brain sample was homogenized using an electric homogenizer at 10,000 rpm in 3 ml of ice-cold RIPA buffer (RIPA Lysis Buffer System; Santa Cruz Biotechnology, Dallas, TX, USA). The homogenate was then centrifuged at 15,000 rpm for 30 minutes at 4°C, and the supernatant was isolated.

The isolated proteins were mixed with 2x Laemmli sample buffer (Bio-Rad Laboratories, Inc., Hercules, CA, USA) plus 2-mercaptoethanol and boiled at 95°C for 5 minutes. For SDS-PAGE, 20 μg of the proteins was loaded into each well of 5–20% gradient gels (ATTO, Tokyo, Japan). The proteins separated on the gel were transferred onto a polyvinylidene difluoride (PVDF) membrane (Merck Millipore, Darmstadt, Germany). For blocking, the PVDF membrane was incubated in 5% skim milk dissolved in PBS-T for one hour at room temperature. To detect CHP1, NHE1 [3], calbindin D-28k, and neurofilament, the proteins were probed with the primary antibodies shown in **Supplementary Table 1**. HRP-conjugated secondary antibodies (Polyclonal Goat Anti-Mouse Immunoglobulins/HRP P0447; Polyclonal Goat Anti-Rabbit Immunoglobulins/HRP P0448; Agilent Technologies) were used, and the chemiluminescence reaction was performed using Immobilon

(Merck Millipore). The signal was visualized and analyzed by densitometry using a LAS4000 mini system (GE Healthcare, Chicago, IL, USA). After analysis, the PVDF membrane was incubated in Stripping Solution (FUJIFILM Wako Pure Chemical Corporation, Osaka, Japan) for 20 minutes at room temperature and then re-probed with the GAPDH antibody as a loading control.

Case presentation

Patient 1

A 30-year-old Japanese male (patient 1) whose parents had been first cousins, and his younger sister who showed similar symptoms (patient 2), presented with ataxic speech and gait, which worsened gradually. No other family members showed similar disorders. Patient 1 had exhibited normal physical and neurological development. At the age of 52 years, however, he developed cognitive decline, depression and delusion. Three years later, he developed walking difficulty and was admitted to a hospital. Neurological examination revealed slurred speech, ocular dysmetria with horizontal nystagumus, limb and trunk ataxia, cognitive decline (WAIS-R: IQ 34) and loss of deep tendon reflexes. The Romberg sign was present. Nerve conduction studies revealed no abnormal findings except for absence of H waves. A fundus examination revealed no abnormality, but a hearing test demonstrated severe bilateral hearing loss. The patient was diagnosed as having spinocerebellar ataxia. Rehabilitation therapy improved his walking ability, and he was discharged. Thereafter, however, his condition slowly deteriorated. At the age of 62 years, he demonstrated bulbar palsy, urinary incontinence and extensor plantar reflexes as well as cerebellar ataxia, and became wheelchair bound. Brain CT revealed diffuse cerebellar atrophy. Three years later, mental decay occurred and he became bed-ridden. At the age of 66 years, he died of melena due to gastric cancer.

Patient 2

This patient was a younger sister of patient 1. She had suffered mild mental retardation and walking difficulty from childhood. At the age of 56 years, her gait disturbance worsened and she became wheelchair bound, accompanied by cognitive decline (HDS-R: 17/30). No Romberg sign was detected. Five years later, a medical examination revealed intellectual disability, slurred speech, ocular dysmetria with vertical nystagmus, ataxic gait, hearing loss, spastic paralysis of a lower limb and extensor plantar reflex. A high palatal arch was evident. Brain CT demonstrated diffuse cerebellar atrophy (**Supplementary figure 1**). At the age of 66 years, she developed irritability and urinary incontinence, followed by severe cognitive decline (WAIS-R: IQ 30). Protirelin tartrate hydrate was prescribed for the gait disturbance, and was partially effective. At the age of 76 years, she died of pneumonia.

General autopsies of these siblings were performed. The brains showed no pathological

features suggestive of complications arising from Alzheimer's disease or Parkinson's disease.

Supplementary references

1. Cingolani P, Platts A, Wang LL, Coon M, Nguyen T, Wang L et al (2012). A program for annotating and predicting the effects of single nucleotide polymorphisms, SnpEff: SNPs in the genome of Drosophila melanogaster strain w1118; iso-2; iso-3. Fly (Austin) 6:80–92. http://www.ncbi.nlm.nih.gov/pubmed/22728672. Accessed 1 Apr 2012.
2. Li H (2013). Aligning sequence reads, clone sequences and assembly contigs with BWA-MEM. https://arxiv.org/abs/1303.3997. Accessed 26 May 2013.
3. Liu Y, Zaun HC, Orlowski J, Ackerman SL (2013). CHP1-mediated NHE1 biosynthetic maturation is required for Purkinje cell axon homeostasis. J Neurosci 33:12656-69.
4. McKeith IG, Boeve BF, Dickson DW, Halliday G, Taylor JP, Weintraub D et al (2017). Diagnosis and management of dementia with Lewy bodies: fourth consensus report of the DLB Consortium. Neurology 89:88-100.
5. Monine TJ, Phelps CH, Beach TG, Bigio EH, Cairns NJ, Dickson DW et al (2012). National institute on Aging-Alzheimer's association guidelines for the neuropathologic assessment of Alzheimer's disease: a practical approach. Acta Neuropathol 123:1-11.
6. Paila U, Chapman BA, Kirchner R, Quinlan AR (2013). GEMINI: integrative exploration of genetic variation and genome annotations. PLoS Comput Biol 9:e1003153. http://www.ncbi.nlm.nih.gov/pubmed/23874191. Accessed 18 Jul 2013.
7. Richards S, Aziz N, Bale S, Bick D, Das S, Gastier-Foster J et al (2015). Standards and guidelines for the interpretation of sequence variants: a joint consensus recommendation of the American College of Medical Genetics and Genomics and the Association for Molecular Pathology. Genet Med 17:405-424.
8. Van der Auwera GA, Carneiro MO, Hartl C, Poplin R, Del Angel G, Levy-Moonshine A et al (2013). From FastQ data to high confidence variant calls: the Genome Analysis Toolkit best practices pipeline. Curr Protoc Bioinforma 43:11.10.1-11.10.33. http://www.ncbi.nlm.nih.gov/pubmed/25431634. Accessed 15 Oct 2013.

Supplementary figure 1. Brain CT images of patient 2

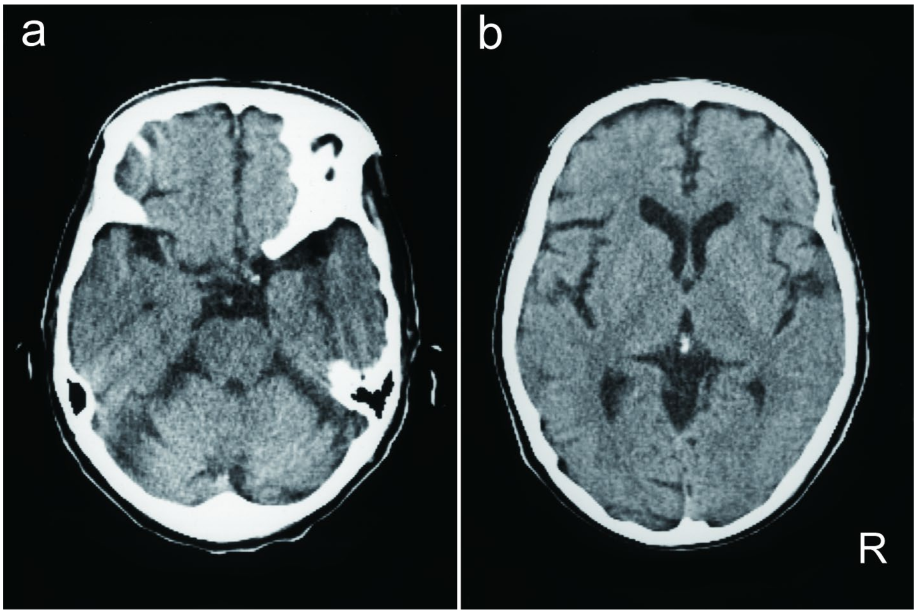

Brain CT images of patient 2 at the age of 63 years. Diffuse atrophy of the cerebellum is evident (**a**), but the cerebrum, basal ganglia and brainstem show no apparent atrophy (**b**). R: The right side of the brain.

Supplementary table 1. Primary antibodies

Antigen (clone)	Antibody species	Source	Dilution	Antigen retrieval
<u>Immunohistochemistry</u>				
Calbindin-D28k (CB300)	Mouse	Swant, Fribourg, Switzerland	1:50	Microwave
Parvalbumin (PARV-19)	Mouse	Sigma Aldrich, St. Louis, MO, USA	1:2000	None
CHP1 (OTI4B9)	Mouse	Thermo Fisher, Rockford, IL, USA	1:1000	Microwave
p62 (3/P62)	Mouse	BD, San Jose, CA, USA	1:1000	Microwave
Polyglutamine (5TF1-1C2)	Mouse	Chemicon, Temecula, CA, USA	1:16000	Formic acid
Amyloid β (11-28, 12B2)	Mouse	IBL, Gunma, Japan	1:50	Formic acid
Phosphorylated tau (AT8)	Mouse	Fujirebio, Ghent, Belgium	1:200	None
Phosphorylated α-synuclein (pSyn#64)	Mouse	Wako, Saitama, Japan	1:1000	Formic acid
<u>Western blot</u>				
CHP1 (OTI4B9)	Mouse	Thermo Fisher, Rockford, IL, USA	1:500	None
NHE1 (polyclonal)	Rabbit	GeneTex, Irvine, CA, USA	1:4000	None
Calbindin-D28k (CB300)	Mouse	Swant, Fribourg, Switzerland	1:500 (frontal), 1:2000 (cerebellum)	None
Neurofilament-H (RmdO20.11)	Mouse	Thermo Fisher, Rockford, IL, USA	1:1000 (frontal), 1:100 (cerebellum)	None
GAPDH (B2534M)	Mouse	Meridian Life Science Inc., Memphis, TN, USA	1:100000	None

Supplementary table 2. Profiles of compound heterozygous candidate variants segregating in the studied patients

Chr	Start	End	dbSNP	Ref	Alt	Gene	Transcript	Exon	Codon change	Amino acid change	Impact	Max_aaf _all (%)	ToMMo aaf (%)	CADD	Cbll
19	11,943,335	11,943,336	rs373205945	A	G	*ZNF440*	NM_152357.2	4/4	c.1345A>G	p.Ile449Val	missense	0.012	0.060	5.8	7.59
19	11,943,373	11,943,374	rs145666235	G	C	*ZNF440*	NM_152357.2	4/4	c.1383G>C	p.Lys461Asn	missense	0.200	0.680	2.9	7.59
2	185,801,096	185,801,097	rs139826553	G	T	*ZNF804A*	NM_194250.1	4/4	c.974G>T	p.Cys325Phe	missense	0.400	0.680	12.8	5.88
2	185,802,171	185,802,172	None	T	TTA	*ZNF804A*	NM_194250.1	4/4	c.2051_2052dupAT	p.Asp685fs	frameshift	-	-	None	5.88

Four segregated candidate variants causing protein alteration and showing a low allele frequency of < 1% and gene expression > 1 median transcripts per kilobase million (TPM) in the cerebellum.. None of the candidates demonstrated a causal link to ARCAs. Variants were annotated using *snpEff* 4.3i and *gemini* 0.19.1. The genomic positions of the variants are based on hg19, and *Start* and *End* represent the 0-based and 1-based genomic position, respectively. *Impact* shows the biological consequence of the most severely affected transcript. *Max_aaf_all* shows the maximum alternate allele frequency in each population from the 1000 Genomes Project, Exome Sequencing Project, or Exome Aggregation Consortium. *ToMMo aaf* shows the alternate allele frequency in 3,554 Japanese whole genomes obtained from Tohoku University Tohoku Medical Megabank Organization. *CADD* shows the PHRED-like scaled scores of predictive deleteriousness, and typically scores of 10 or higher indicate possibly pathogenic variants. The column labeled *Cbll* represents the amount of gene expression in the cerebellum based on GTEx Portal V8. The values reperesent the median TPM.
